# Discovery and characterization of a new bacterial candidate division by an anaerobic sludge digester metagenomic approach

**DOI:** 10.1111/j.1462-2920.2008.01632.x

**Published:** 2008-08

**Authors:** Sonda Guermazi, Patrick Daegelen, Catherine Dauga, Delphine Rivière, Théodore Bouchez, Jean Jacques Godon, Gábor Gyapay, Abdelghani Sghir, Eric Pelletier, Jean Weissenbach, Denis Le Paslier

**Affiliations:** 1 CEA/Genoscope91057 Evry, France; 2 CNRS-UMR 803091057 Evry, France; 3 Université d'Evry Val d'Essonne91057 Evry, France; 4 Plateforme 4, Génopole de l'Institut PasteurParis, France; 5 Suez-Environment CIRSEE78230 Le Pecq, France; 6 Cemagref, Hydrosystèmes et Bioprocédés, Parc de TourvoieBP 44, 92163 Antony cedex, France; 7 INRA, UR050, Laboratoire de Biotechnologie de l'EnvironnementNarbonne, F-11100, France

## Abstract

We have constructed a large fosmid library from a mesophilic anaerobic digester and explored its 16S rDNA diversity using a high-density filter DNA–DNA hybridization procedure. We identified a group of 16S rDNA sequences forming a new bacterial lineage named WWE3 (Waste Water of Evry 3). Only one sequence from the public databases shares a sequence identity above 80% with the WWE3 group which hence cannot be affiliated to any known or candidate prokaryotic division. Despite representing a non-negligible fraction (5% of the 16S rDNA sequences) of the bacterial population of this digester, the WWE3 bacteria could not have been retrieved using the conventional 16S rDNA amplification procedure due to their unusual 16S rDNA gene sequence. WWE3 bacteria were detected by polymerase chain reaction (PCR) in various environments (anaerobic digesters, swine lagoon slurries and freshwater biofilms) using newly designed specific PCR primer sets. Fluorescence *in situ* hybridization (FISH) analysis of sludge samples showed that WWE3 microorganisms are oval-shaped and located deep inside sludge flocs. Detailed phylogenetic analysis showed that WWE3 bacteria form a distinct monophyletic group deeply branching apart from all known bacterial divisions. A new bacterial candidate division status is proposed for this group.

## Introduction

A limiting step in understanding any microbial ecosystem resides in our ability to inventory the microorganisms inhabiting the ecosystem, and to assess their metabolic potential, the interactions between them and their biotope. A partial answer to this challenge was (i) culture-independent studies based on the development of molecular microbial diversity analyses using the 16S rDNA gene as a phylogenetic marker (Olsen *et al*., 1985; Woese, 1987; Ludwig and Schleifer, 1999), and (ii) the development of metagenomic studies of complex ecosystems. Large-scale sequencing efforts in various ecosystems such as a community from acid mine drainage (Tyson *et al*., 2004; Baker *et al*., 2006), the Sargasso Sea (Venter *et al*., 2004) and the Global Ocean Sampling expedition (Rusch *et al*., 2007) have considerably enriched our understanding of uncultured microbial communities. These studies made it possible to link phylogeny and function, revealing a surprising abundance of different types of genes, and enabled the reconstruction of genomes of organisms that have not been cultured to date (Erkel *et al*., 2006).

Anaerobic sludge digesters are complex ecosystems in which a consortium of microorganisms degrades organic matter into methane and carbon dioxide under anaerobic conditions. The organisms involved are still awaiting species diversity and metabolic characterization. Recent studies of the diversity of wastewater microbial communities based on the analysis of 16S rDNA sequences have extended our knowledge about the diversity of this ecosystem (Godon *et al*., 1997; Chouari *et al*., 2003; 2005a,b). Moreover, the recent discovery of the new WWE1 (Waste Water of Evry 1) candidate division by molecular inventories of the anaerobic digester of Evry (Chouari *et al*., 2005b) has shown that additional bacterial and archaeal populations remain to be described.

In the present study, we report that in the course of analysing a metagenomic fosmid library constructed from an anaerobic digester, we detected an unusual group of 16S rDNA bacterial gene sequences. These sequences, which presented many mismatches with the 16S rDNA universal primers, cannot be obtained by the classical polymerase chain reaction (PCR)-based amplification methods. Specific 16S rDNA PCR primers were developed for this group of sequences, named WWE3. Phylogenetic analyses show that this group constitutes a new bacterial candidate division. Fluorescence *in situ* hybridization (FISH) experiments helped to provide information about morphology and localization of the WWE3 bacteria within a microbial anaerobic sludge sample. Furthermore, the absence of the H17 helix in the WWE3 16S rDNA secondary structures is unprecedented and seems to be a characteristic of the bacterial candidate division WWE3 and its closest relatives.

## Results

### Metagenomic clone library construction and screening, fosmid sequencing and primer design

In order to analyse the microbial diversity and the metabolic potential of a mesophilic anaerobic digester, a large fosmid library was constructed using DNA extracted from the sludge digester of the WWTP of Evry, France. A part of the library (27 648 fosmid clones) was screened by hybridization with 16S rDNA gene targeted-hybridization probes. The 16S rDNA genes of 570 positive clones were directly sequenced using internal primers. While for 541 of these positive clones, the 16S rDNA gene sequences were obtained and affiliated to known bacterial or archaeal phyla, we were unable to obtain a 16S rDNA sequence for 29 clones. Analysis of HindIII fingerprints of these clones showed that their profiles were very similar. Southern blot hybridization using 16S rDNA-targeting probes revealed that 27 out of the 29 clones showed a common 1.6 kb HindIII positive fragment while the remaining two clones possess a positive 1.65 kb fragment. Shotgun sequencing of one of these 29 fosmid clones (DIGA11YD11) revealed that it does contain a complete 16S rDNA gene sequence which affiliates (88% identity) with a single sequence (AY953190) in public databases. The 16S rDNA sequences of the remaining 28 fosmids were determined by direct sequencing with specific primers derived from the DIGA11YD11 16S rDNA (Table 1). All these 16S rDNA gene sequences share more than 99% identity. Two of them (fosmids DIGA75YB16 and DIGA43YA13; corresponding to those presenting the 1.65 kb positive HindIII hybridization fragment) have a 65 bp insertion (type I insertion). The 29 16S rDNA gene sequences present at least two mismatches with the commonly used 16S rDNA PCR and sequencing primers used in the study (Table 2). The presence of these mismatches could explain the failure to obtain their 16S rDNA sequence as well their absence in public databases.

**Table 2:** Mismatches between the DIGA11YD11 16S rDNA sequence and PCR and sequencing primers.

PCR and sequencing primers	Sequence 5′−3′^a^
ACM_1517R^b^	
DIGA11YD11	
ADM_1110R^b^	
DIGA11YD11	
SSM_8F^b^	
DIGA11YD11	
DIGA11YD11	
TTM_330F (EUB_338_I)^b^	
EUB_338_II	
EUB_338_III	
Univ-1390R	
DIGA11YD11	

a Dashes indicate identity with the homologous nucleotides in the target sequence.

b Sequencing primers used in this study (Pelletier *et al*., 2008).

**Table 1:** Summary of PCR primers and combinations used for WWE3 detection and 16S rDNA library construction.

				Primer sets						
Specificity	Primer	Sequence 5′−3′	Position^a^	1	2	3	4	5	6	7
Bacteria	Bact-008F^b^	AGAGTTTGATCCTGGCTCAG	0008−0027						+	
Universal	Univ-1390R^b^	GACGGGCGGTGTGTACAA	1407−1390					+		
	WWE3-ExtF	GCACTTTGAAAAGGTATCCT		+						
	WWE3-ExtR	CCTACTCAACTGTTTGTGAG		+						
	WWE3-21F	GGTTCAGGGTGAATGCTA	0021−0038		+			+		
Candidate	WWE3-1322R	CTTTGCTGACGTGACGGG	1402−1419		+				+	
Division	WWE3-289F	GGGCACTGAGACACGGG	0317−0334				+			
WWE3	WWE3-948R	TGGATACCGGTCGTTCC	1031−1019				+			
	WWE3-149F	GGCGGGGTAATTCCTTAT	0163−0180			+				
	WWE3-1202R	CTGAGAGGTCGTTTAGCG	1300−1282			+				
	WWE3-21DF	GGNTCAGGGTGAATGCTA	0021−0038							+
	WWE3-1282DR	CRTATTCACSGNNGTATAGCTG	1380−1359							+

a Position of the primers was determined in reference to *E. coli* sequence (A14565).

b All primers used were designed in the study, except Bact-008F (Hicks *et al*., 1992) and Univ1390R (Zheng *et al*., 1996).

Annealing temperature for all primer sets was 57°C, except for set 7 (53°C).

Primer sets 1, 2, 3 and 4 were used for PCR screening of environmental samples for the presence of WWE3 representatives. Primer sets 1, 2, 5, 6 and 7 were used for 16S rDNA library construction.

### The extent and diversity of WWE3 representatives

In order to investigate the presence and the diversity of the WWE3 phylogenetic group, specific PCR primers targeting different regions of the DIGA11YD11 16S rDNA were designed (Table 1). A total of 64 different DNA samples (Table 3 and *Experimental procedures*) were tested using the four DIGA11YD11-specific primer sets 1–4 (Table 1). Polymerase chain reaction amplification products were obtained from 20 anaerobic digesters located in different countries in Europe and America as shown in Table 3. Cloning and sequencing of part of these PCR products confirmed their affiliation to the WWE3 group (see bottom).

**Table 3:** Characteristics of the anaerobic digesters that were tested for the presence of WWE3 bacteria.

Country	Digesters	WWE3^a^	Scale^b^	Process^c^	Effluent^d^
Canada	Montreal	−	I	CST	W
	Montreal	−	L	UASB	Phenolic compounds
Chile	El Trebal	−	I	CST	W
Czech Republic	Brno	−	I	CST	W + 30% dairy, food industry
	Zabreh	−	I	CST	W + 30% dairy, food industry
France	Aix en Provence I, II	−	I	CST	W
	Asnières sur Oise	+	I	CST	W
	Carré de la réunion	+	NA	NA	NA
	Cholet^e^	+	I	CST	W + 9% slaughterhouse
	Clos de Hilde	−	I	CST	W
	Conneré	+	I	FBR	Cassoulet and sauerkraut
	Corbeil^e^	+	I	CST	W
	Creil^e^	+	I	CST	W
	Evry^e^	+	I	CST	W
	Haguenau	−	I	CST	W + 30% mechanical, food industry
	La Roche sur Foron	−	I	CST	W + 50% food industry
	Les Mureaux	−	P	CST	W
	Marseille	−	I	CST	W
	Marseille	−	I	CST	W
	Montardon	−	P	SBR	Pig slurry
	Narbonne	−	L	SBR	Pig slurry
	Narbonne	−	L	SBR	Pig slurry
	Narbonne	+	L	UASB	Lignin
	Narbonne	−	P	FBR	Vinasses
	Narbonne	−	L	SBR	Vinasses
	Rochefort	−	I	CST	W + 12% industry, heavy metals
	St.Laurent de Cognac	−	I	FB	Acidogenic vinasses
	St.Laurent de Cognac	−	I	FB	Acidogenic vinasses
	St.Laurent de Cognac	+	I	CST	Lees vinasses
Germany	Goslar	+	I	CST	W
	Manheim^e^	+	I	CST	W + 50% paper industry
	Mulheim	−	I	CST	W + green wastes
	Rostock	+	I	CST	W + 28% food industry
Ireland	Cork	+	I	UASB	Citric acid from beet molasses production
Italy	Casolino I,^e^ II	+	I	CST	W + 15% industry, heavy metals
Mexico	Culiacan	−	I	CST	W
	Mexico city	+	L	UASB	Rum vinasses
	Mexico city	−	L	FBR	Rum vinasses
	Mexico city	−	L	UASB	Yeast factory
	Puebla	−	I	CST	W + 20% textile, colouring industry
Spain	Blanes	−	I	CST	W
	Hoya de Lorca	−	I	CST	W + 70% industrial heavy metals, oil, greases
	Palencia^e^	+	I	CST	W + food industry
	Roquetas de mar	−	I	CST	W + 15% paper industry
	Vic^e^	+	I	CST	W + 40% industry, heavy metals
Switzerland	Bilten	+	I	CST	W + 40% paper, food, textile industry
UK	Stressholme	−	I	CST	W

a Polymerase chain reaction detection of WWE3 bacteria was performed using the DIGA11YD11-specific primer sets 1, 2, 3 and 4.

b I: industrial scale; L: laboratory scale; P: pilot scale.

c CST, continuously stirred tank, FB, fixed biofilm, FBR, fluidized bed reactor, UASB, up-flow anaerobic sludge blanket.

d W, wastewater.

e Used for 16S rDNA library construction.

All the digesters operated at mesophilic temperature, usually 37°C, except Zabreh and one of the digesters of Aix-en-Provence which operates at thermophilic conditions. The Casolino I sample was obtained when the digester was operating at 37°C and sample II after the switch of the temperature to 31°C.

NA, not available.

To explore the extent of diversity of this novel group of 16S rDNA sequences, we proceeded in two steps: (i) 16S rDNA libraries were constructed from DNA extracted from seven anaerobic digesters, using the DIGA11YD11-specific primers or a combination of specific and universal primers, (ii) sequence analysis of these 16S rDNA confirmed their inclusion in the WWE3 phylogenetic group and allowed us to design degenerate primers with a broader specificity. The presence of WWE3 bacteria was further investigated and confirmed in other anaerobic or anoxic environments such as swine lagoon slurries (6/10 samples) and freshwater biofilms (6/6 samples). The presence of WWE3 bacteria was tested by PCR on DNA sampled from three digesters over a 6-year period (2000–2006). During this period, WWE3 bacteria were detected in 16 out of 23 Evry sludge samples, three out of six from Corbeil and in all three sludge samples from Creil, showing that the WWE3 population size is subject to large variations in these anaerobic digesters.

### Clone library analysis

**Fig. 1:**
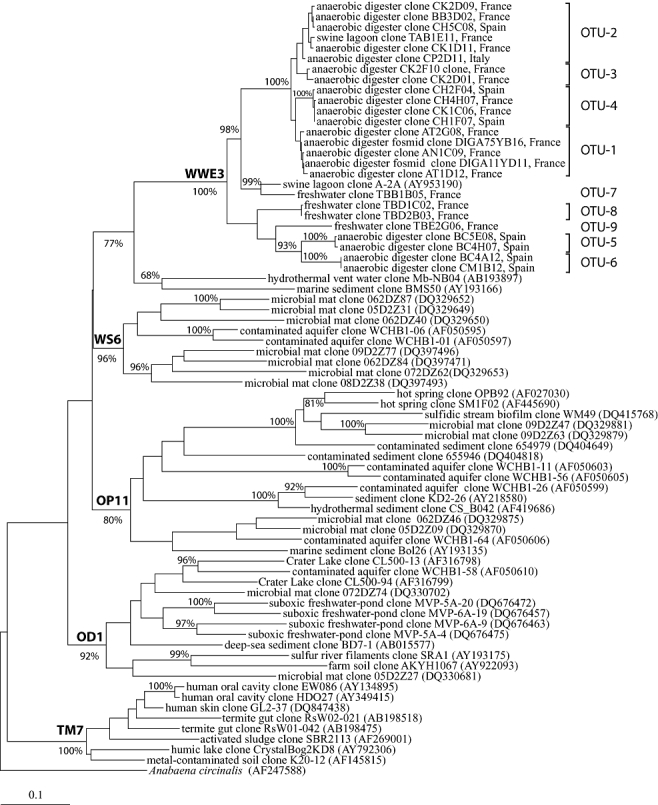
Maximum-likelihood phylogenetic tree showing the relationship of the environmental WWE3 sequences to representatives of the OP11, WS6, OD1 and TM7 divisions. Sequences were aligned with the ARB database and software package. Aligned sequences were analysed by three methods (BioNJ, maximum likelihood and maximum parsimony) provided by PAUP 4.0b10 as described in the text. A total of 1176 homologous positions was used for tree construction. The numbers at the nodes indicate the percentage of recovery of relevant branch points in 100 bootstrap re-samplings. The *Anabaena circinalis* 16S rDNA sequence was used as the outgroup to define the root of the tree. The scale bar represents the 10% estimated difference in nucleotide sequence positions.

A total of 21 16S rDNA libraries were constructed from 12 DNA samples extracted from eight anaerobic digesters (Table 3), one swine lagoon and three freshwater biofilm samples, using different primer sets (sets 1, 2, 5, 6 and 7, Table 2). A total of 1639 sequences were imported into the ARB database (Ludwig *et al*., 2004) and analysed. These sequences were grouped into nine operational taxonomic units (OTU), using a 97% identity cut-off (Stackebrandt and Goebel, 1994) (Fig. 1). Among these OTUs, we noticed that OTU-1 encompasses 91% of the sequences obtained in the study and that representatives of this OTU were recovered exclusively from anaerobic sludge digesters, with all the PCR primer sets used. A 65 bp insertion (type I) was found within 28% of the OTU-1-related 16S rDNA sequences.

An additional 61 bp insertion (type II) was found in the OTU-4 sequences. The two insertions (I and II) are located within the same region but exhibit completely different sequences. The overall intradivergence between WWE3 16S rDNA gene sequences reached 20% and is in the same order of magnitude as that for other uncultured bacterial candidate divisions [e.g. 29% for the OP11 candidate division, 27% for the OD1 candidate division and 15% for the SR1 candidate division (Harris *et al*., 2004)].

### Phylogenetic analysis

In order to solve the WWE3 phylogenetic position, phylogenetic analysis were conducted using 16S rDNA sequences representing the major bacterial divisions, along with WWE3 sequences. Results show that, independently of the method used for tree construction (distance, parsimony and maximum likelihood using ARB, PAUP and PhyML software) and beyond the number of sequences included in the analysis, WWE3 sequences form a monophyletic group, branching distinctly apart from all bacterial and archaeal divisions, with OP11 and WS6 candidate divisions as their closest relatives (data not shown).

In order to refine the WWE3 position, phylogenetic analyses were carried out using representatives of the nine WWE3 OTUs and representatives of OP11, WS6, OD1 and TM7 bacterial candidate divisions. Beyond the method used for tree construction, WWE3 sequences always form a monophyletic group within the OP11-WS6-OD1 cluster (Fig. 1).

The WWE3 division encompasses the swine lagoon clone AY953190 and the partial 16S rDNA gene sequence (713 bp) of the ‘uncultured archaeon’ clone AJ556482 (Wu *et al*., 2006) (this result was supported by a bootstrap value of 100%, data not shown). Based on conducted phylogenetic analysis, we proposed the affiliation of the partial 16S rDNA gene sequence of clone AJ556482 to the WWE3 candidate division. AY953190 and AJ556482 shared an average of 88.0% and 82.4% sequence identity, respectively, with WWE3 representatives. The sequences AB193897 and AY193166 showed 76% average identity with WWE3 sequences and corresponded to the nearest relatives of this division (Fig. 1).

In order to investigate the specific WWE3 branching, another phylogenetic analyses was undergone using the *radA* gene found in the DIGA11YD11 insert. As previously shown, *recA* and *radA* (subfamily member of the *recA* group) can be used as valid gene markers for bacterial and archaeal phylogeny (Eisen, 1995; Sandler *et al*., 1999). Results showed that WWE3-*radA* gene clusters with the bacterial *radA* genes (Fig. S1), but did not belong to any recognized bacterial division.

### Ribosomal RNA secondary structures

**Fig. 2:**
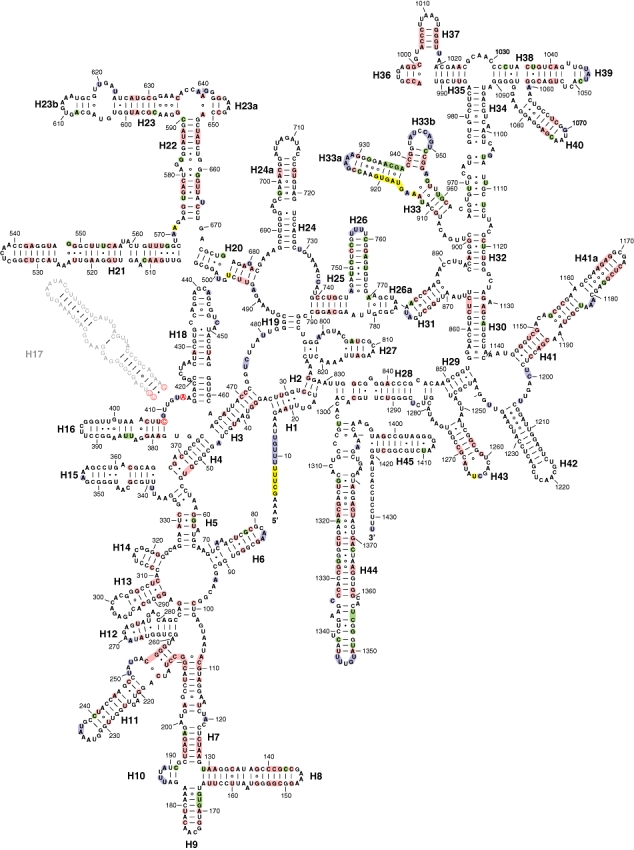
WWE3 DIGA11YD11 16S rDNA secondary structure. This planar structure was determined using the 16S rRNA secondary structure from *E. coli rrsA* as a reference. Coloured spots indicate nucleotides not conserved between the two secondary structures: yellow, supplementary nucleotides; pink, both nucleotides of a base pair are different; green, only one nucleotide of the base pair is different; blue, loop nucleotide variants. The H17 stacked helix from *E. coli rrsA* is represented in grey. The first two nucleotides U437 and U438 and the last G497 of H17 (represented and circled in pale red) could correspond to nucleotides U410, U411 and G412 of DIGA11YD11, while the nucleotides C408 and G414 in DIGA11YD11 (represented and circled in red) probably correspond to nucleotides C436 and A498 in *E. coli rrsA*, in 5′ and 3′ of the H17 helix respectively. Tertiary interactions supported by strong comparative data (RDPII) are not represented except for the H1/H2 pseudoknot.

Secondary structures of the DIGA11YD11 16S ribosomal RNA (rRNA) as well as one representative of each of the nine WWE3 OTUs were calculated. Except for a limited number of supplementary nucleotides, the overall secondary structure of WWE3 16S rRNA was almost homologous to the archetypal 16S rRNA structure (Gutell *et al*., 1994), characteristic of architecture conservation through evolutionary changes (Fig. 2). When comparing the DIGA11YD11 16S rRNA structure with the archetypal *Escherichia coli* K12 16S rRNA structure (Cole *et al*., 2005), we observed that a large number of covariant modifications affect both bases of a number of stem base pairs rather than single bases alone. Multiple nucleotide loop variations were also observed. Several regions of the structure are clearly less subject to changes than others, mainly H16/H18, H23/H24, H27 and H34 compared with H6 and H10. We observed the absence of the entire H17 helix for all the WWE3 16S rRNA structures. The function of H17, which interacts with the S4 and S16 ribosomal proteins (Brodersen *et al*., 2002; Schuwirth *et al*., 2005), is not clear. Sequence analysis showed that only two 16S rDNA sequences (AY953190 and AB193897) from the RDP release 9.36 lack this H17 helix (data not shown).

The lack of H17 was reported in another sequence, AY193166 (absent from the RDP II database), and classified as a member of the WS6 division by Harris and colleagues (2004). Thus, the absence of this helix appears as a characteristic of all members of WWE3 candidate division and some unclassified closest relatives. The H10 region is characterized for some members of the WWE3 division belonging to OTU-1 and OTU-4 by insertions of type I and II (65 and 61 bp respectively) while the H6 subdomain is extremely variable between WWE3 representatives but is conserved in all structures as a coaxial stacked helix.

### Fosmid annotation

The complete DIGA11YD11 clone sequence was obtained using standard shotgun strategy. The 39 kb DIGA11YD11 fosmid presents a low percentage of G+C (36.13%). Thirty-one predicted protein-coding sequences and five RNA-coding genes were annotated. Some of the predicted genes seem to be directly related to DNA or RNA metabolism and also to known enzymatic functions involved in glucose metabolism and membrane transport; the others correspond to hypothetical proteins (Table 4). No conclusions regarding specific metabolism of WWE3 organisms indicating their possible role in the anaerobic sludge digestion could be inferred from the annotation of this fosmid.

**Table 4:** Annotation of the DIGA11YD11 predicted genes using the MaGe annotation system.

Label	Begin	End	Gene	Product	EC number	Cellular role
WWE3-TFM_1	58	2190		Putative UvrD/REP helicase		DNA metabolism
WWE3-TFM_2	2202	3302		Putative DNA recombination protein		DNA metabolism
WWE3-TFM_3	3337	3702		Hypothetical protein		Unknown function
WWE3-TFM_4	3781	5196	*metG*	Methionyl-tRNA synthetase (Methionine-tRNA ligase) (MetRS)	6.1.1.10	Protein synthesis
WWE3-TFM_5	5214	6134		Putative 5′−3′ exonuclease		DNA metabolism
WWE3-TFM_6	6208	6861	*nth*	Endonuclease III [DNA-(apurinic or apyrimidinic site) lyase]	4.2.99.18	DNA metabolism
WWE3-TFM_7	6882	7493		Hypothetical protein		Unknown function
WWE3-TFM_8	7537	8484		Putative sugar kinase		Unknown function
WWE3-TFM_9	8487	9485		Putative transketolase C-terminal section (TK)	2.2.1.1	Central intermediary metabolism
WWE3-TFM_10	9507	10115		Hypothetical protein		Unknown function
WWE3-TFM_11	10120	10968		Putative transketolase N-terminal section (TK)	2.2.1.1	Central intermediary metabolism
WWE3-TFM_12	11277	12545		‘Multifunctional protein [Ribulose-phosphate 3-epimerase; unknown domain]’	5.1.3.1	Central intermediary metabolism
WWE3-TFM_13	12555	13004		Putative ribose-5-phosphate isomerase B (Phosphoriboisomerase B)	5.3.1.6	Central intermediary metabolism
WWE3-TFM_14	13001	13753	*lgt*	Prolipoprotein diacylglyceryl	2.4.99.-	Protein fate transferase
WWE3-TFM_15	13770	14351		Hypothetical protein		Unknown function
WWE3-TFM_16	14547	14837		Hypothetical protein		Unknown function
WWE3-TFM_17	14870	16672		Putative DNA ligase	6.5.1.1	DNA metabolism
WWE3-TFM_18	16719	17711		Conserved hypothetical protein		Unknown function
WWE3-TFM_19	18223	19281	*recF*	DNA replication and repair protein RecF		DNA metabolism
WWE3-TFM_20	19306	20187	*mutM*	Formamidopyrimidine-DNA glycosylase (Fapy-DNA glycosylase) [DNA-(apurinic or apyrimidinic site) lyase mutM] (AP lyase mutM)	3.2.2.23, 4.2.99.18	DNA metabolism
WWE3-TFM_21	20180	21304		Putative glycosyltransferase		Unknown function
WWE3-TFM_22	21407	21919		Hypothetical protein		Unknown function
WWE3-TFM_23	22015	22503		Hypothetical protein		Unknown function
WWE3-TFM_24	22518	24944		Hypothetical protein		Unknown function
WWE3-TFM_25	25073	26404		Conserved hypothetical protein		Unknown function
WWE3-TFM-r1	26575	28006		16S rRNA		Protein synthesis
WWE3-TFM_26	28621	29046		Putative NUDIX hydrolase		Unknown function
WWE3-TFM_27	29132	29761		Hypothetical protein		Unknown function
WWE3-TFM_28	29746	29997		Conserved hypothetical protein		Unknown function
WWE3-TFM-t1	30580	30655		tRNA-Ile		Protein synthesis
WWE3-TFM_29	30876	31865		Conserved hypothetical protein		Unknown function
WWE3-TFM-t2	32101	32177		tRNA-Ala		Protein synthesis
WWE3-TFM-r2	32570	35588		23S rRNA		Protein synthesis
WWE3-TFM-r3	35719	35837		5S rRNA		Protein synthesis
WWE3-TFM_30	36123	37367	*rpsA*	30S ribosomal protein S1		Protein synthesis
WWE3-TFM_31	37367	38689	*radA*	DNA repair protein radA homologue (DNA repair protein sms homologue)		DNA metabolism

### FISH experiments

**Fig. 3:**
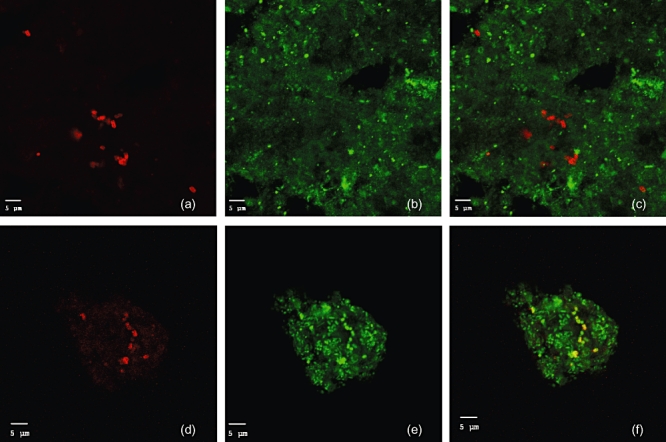
Epifluorescence micrographs of WWE3 bacteria in sludge samples from the anaerobic digester of Evry. A and D. Cy3-labelled DIGA11YD11-21-specific probe (red). B. FITC-labelled Eub338 probe mix (green). C. Colour combination of (A) and (B), WWE3 bacteria were not labelled by the Eub338 mix probe and then did not appear yellow. E. SYTO 9 staining (green). F. Colour combination of (D) and (E), WWE3 bacteria appear yellow.

Fluorescence *in situ* hybridization analysis using the DIGA11YD11-21-Cy3-labelled probe was performed on Evry digester sludge samples. Microscopic observations using a confocal laser scanning microscope revealed that DIGA11YD11-21-Cy3-positive cells are oval-shaped and are usually observed to form aggregates located inside sludge flocs (Fig. 3). No hybridization signals were recorded when nonsense DIGA11YD11-21-Cy3-labelled probe was used as non-specific hybridization control. It should, however, be noted that standard FISH protocols were employed in our experiments, including PFA fixation and centrifugation steps that might have interfered with floc structure.

As expected, no hybridization signal was obtained using the EUB-mix (a mixture of probes EUB-338 I, II and III) as 16S rDNA gene sequences of members of the WWE3 bacteria show at least two mismatches with EUB-338 probes. Superposition of SYTO 9 and WWE3-specific signals showed an unusual ring-shaped localization pattern of the probe-specific hybridization signals, suggesting a hypothetical compartmentalization of the cytoplasm. Interestingly, analogous arrangements have already been reported for members of the *Planctomycetales* (Fuerst, 2005) and *Poribacteria* divisions (Fieseler *et al*., 2004).

The size of the WWE3 population exhibited extensive variation from sample to sample. Moreover, for a given sample, unequal distribution of probe-positive cells from floc to floc was recorded. Overall, the estimated proportion of WWE3 population varied from undetectable levels to up to 5% of the SYTO 9-stained biomass*.*

## Discussion

During the last few decades, rRNA gene sequence comparison has been the classical way of examining microbial diversity in natural environments (Rappé and Giovannoni, 2003). The number of 16S rDNA sequences deposited in the databases is still increasing very rapidly, as well as the number of archaeal and bacterial phyla. These 16S rDNAs were affiliated with almost one hundred bacterial divisions [Greengenes database (http://greengenes.lbl.gov) (DeSantis *et al*., 2006)]. More recently, metagenomic approaches applied to the study of environmental samples have led to the discovery of novel microorganisms with an important role in the biological carbon and nitrogen cycles (Treusch *et al*., 2005; Hallam *et al*., 2006; Leininger *et al*., 2006). A similar metagenomic approach permitted the discovery of a new archaeal division that was not previously detected when using classical primer sets (Baker *et al*., 2006). The primers usually used to obtain almost full-length sequences were designed on the basis of rDNA sequences from cultured organisms (Weisburg *et al*., 1991). However, a number of phylogenetic groups remain undetected because they show more than one mismatch with the commonly used PCR primers (Baker *et al*., 2003).

In this study, we tested whether new bacterial phyla could be discovered by a metagenomic approach using a DNA–DNA hybridization procedure. This approach, based on the sequence identity and not on PCR, allowed us to find several clones bearing a 16S rDNA representative of a newly defined bacterial candidate division we named WWE3. These WWE3 16S rDNA sequences have at least two mismatches with the commonly used PCR primers and probes, explaining in part the reason behind the quasi-absence of representatives of this group in public databases.

According to commonly accepted criteria (Hugenholtz *et al*., 1998; Rappé and Giovannoni, 2003; Harris *et al*., 2004), the WWE3 group of sequences constitutes a new bacterial candidate phylum: (i) more than three distinct WWE3 sequences were obtained from independent PCR products, (ii) WWE3 sequences are of a minimum of 1 kb in length, and (iii) phylogenetic analysis of more than 1600 nearly full-length 16S rDNA gene sequences retrieved from 12 different environmental samples showed that WWE3 sequences, when compared with representatives of known bacterial phyla, form a monophyletic group branching apart from the other bacterial divisions, with a percentage of intradivergence of 20% [the cut-off used to distinguish a new bacterial phylum being 85% according to Hugenholtz and colleagues (1998)].

Apart from WWE3 sequences, H17 deletion was only observed in sequences AB193897 and AY193166, for which affiliation is unclear.

Polymerase chain reaction screening using WWE3-specific primers documented the diversity of this bacterial candidate division and permitted the detection of bacteria from this group in a number of different ecosystems (anaerobic sludge digesters in many different locations worldwide, swine lagoon slurries and freshwater biofilms). The use of additional WWE3-PCR primers to test other terrestrial and aquatic environments may enable us to have a broader view of this phylum and provide some hints on its metabolic lifestyle.

*In situ* hybridization indicated that WWE3 bacteria were embedded in sludge flocs and that they may present cytoplasmic compartmentalization as has been shown for some members of the *Planctomycetales* (Fuerst, 2005) and *Poribacteria* (Fieseler *et al*., 2004). Study of this compartmentalization by transmission electron microscopy will require isolation of WWE3 representatives.

In the studied sample, WWE3 has been identified as a significantly abundant group of microbes (29 out of the 570 16S rDNA-bearing fosmids) that was undetectable through PCR approach using ‘universal primers’. A metagenomic approach has thus proven to be effective in discovery of yet undescribed microbial groups. With decreasing costs, sequencing may represent a valuable alternative for a nearly exhaustive identification of prokaryotic divisions in natural environments.

## Experimental procedures

### Metagenomic library screening

Construction and screening of part of the fosmid metagenomic library, using genomic DNA extracted from the anaerobic mesophilic digester of Evry (France), was performed as described by Pelletier and colleagues (2008). Briefly, fosmid DNA was extracted from 27 648 clones (384 × 72) and spotted in duplicate onto 20 × 20 cm nylon membranes (Hybond N+, GE Healthcare Europe GmbH, Saclay, France). Membranes were successively hybridized with ^32^P-labelled complex 16S rDNA probes representatives of the different archaeal and bacterial lineages described in the WWTP of Evry (Chouari *et al*., 2003; 2005a,b). Positive clones were picked and their 16S rDNA was directly sequenced using a set of four internal primers (Table 2). For 29 clones, the 16S rDNA sequence was not obtained. HindIII fingerprints of these 29 clones were performed and Southern blot was hybridized with the same 16S rDNA-targeting probes. One of these fosmids, DIGA11YD11, was shotgun sequenced.

### Sample collection

DNA was extracted from 64 different samples and tested by PCR for the presence of WWE3 bacteria. A total of 48 anaerobic sludge digester samples are described in Table 3. Ten swine lagoon sludges as well as six freshwater biofilms were also tested. All freshwater biofilms were obtained from a river (Rû de Balory, close to Evry, France).

### Primer and probe design

The 16S rDNA sequence obtained from the fosmid DIGA11YD11 was used to design PCR primers and FISH probes. All possible 18 bp oligonucleotides were generated and those specific only for the DIGA11YD11 clone were retained. Final PCR primers were checked for their low potential for hairpin formation and FISH probes were chosen by estimating their accessibility to target sites as described (Behrens *et al*., 2003). Characteristics of the PCR primers are described in Table 1. Primer set number 1 was used to determine the 16S rDNA sequences of the 28 fosmids.

### PCR amplification, cloning and sequencing of WWE3 16S rDNA

16S rDNA clone libraries were constructed using DNA extracted from sludge samples obtained from anaerobic digesters in wastewater treatment plants in Cholet, Corbeil, Creil, Evry, Manheim, Palencia and Vic (Table 3), using specific primer sets 1 and 2 and also combinations of DIGA11YD11-specific primers with bacterial and universal primers (sets 5 and 6). The 16S rDNA amplicons were cloned using a TA cloning kit (pGEM-T vector; Promega) in accordance with the manufacturer's instructions. DNA sequencing was performed using standard methods. Analysis (alignments and secondary structures) of these 16S rDNA gene sequences allowed us to design degenerate primers (set 7) targeting all the known WWE3 16S rDNA sequences. These newly designed degenerate primers were used for 16S rDNA clone library construction using DNA extracted from one swine lagoon sample, three freshwater biofilms and five sludge samples (Casolino, Cholet, Manheim, Palencia and Vic, Table 3).

### Phylogenetic analysis

The 16S rDNA sequences obtained were edited and assembled with Phrap (http://www.phrap.org/). For all subsequent phylogenetic analysis, we used sequences containing at least 1200 nucleotides. The resulting 16S rDNA sequences were chimera checked and then compared with blast to those available in public databases [GenBank, RDP (http://rdp.cme.msu.edu/), and Greengenes (http://greengenes.lbl.gov)]. The retained sequences were then imported into the ARB database (http://www.arb-home.de) for phylogenetic analyses. An automatic alignment was performed which was manually checked and corrected.

WWE3 16S rDNA sequences were compared with 16S rDNA sequences representative of the main bacterial divisions described in public databases and phylogenetic analyses were performed using representatives of these bacterial phyla (data not shown). Twenty-five 16S rDNA sequences representative of WWE3-defined OTUs, as well as representatives of OP11, WS6, OD1 and TM7 candidate divisions were used for tree construction. A modified version of the ‘Lane mask’ was used to choose homologous positions for tree construction (Lane, 1991). Phylogenetic trees were built using three methods provided by PAUP 4.0b10 software (Swofford, 2002): distance (BioNJ), maximum likelihood and maximum parsimony. For all the sequence sets studied, models of nucleotide substitution were evaluated with modeltest 3.0 (Posada and Crandall, 2001) to identify the model that best fit the data. Distance- and maximum likelihood-based phylogenetic trees were constructed with the General Time Reversible (Tavaré, 1986) nucleotide substitution model. The heterogeneity of nucleotide substitution rates among sites was approximated by a gamma distribution and an assumption of invariable sites. Maximum-likelihood analyses were carried out with a heuristic search strategy to find the best trees. The maximum-parsimony trees were built with the full heuristic search and the tree bisection reconnection (TBR) branch-swapping option. A strict consensus tree was drawn when multiple best trees were obtained. Statistical confidence levels for maximum-likelihood, maximum-parsimony and BioNJ trees were evaluated by the non-parametric bootstrap method based on 100 re-samplings. Bootstrap for maximum-likelihood analysis was performed without branch swapping to reduce computational time.

The *radA* annotated gene from DIGA11YD11 fosmid was aligned along with bacterial (sequences subset extracted from family HBG000623) and archaeal and eukaryotic (family HBG049531) *radA* homologues obtained from HOGENOM (http://pbil.univ-lyon1.fr). Phylogenetic analysis were performed using PhyML (Guindon and Gascuel, 2003).

### WWE3 distribution

The presence of the WWE3 bacteria was checked by PCR amplification using DIGA11YD11-specific primer sets 1, 2, 3 and 4, on 23 DNA samples extracted from the anaerobic digester of Evry (recovered from 2000 to 2006), six samples from Corbeil and three from Creil. The other digester samples are described in Table 3. Swine lagoon and freshwater biofilm samples were tested using specific primer sets and degenerate primers (set 7).

### FISH experiments

Sludge aliquots from the anaerobic digester of Evry were prepared for FISH experiments by washing in PBS and then by paraformaldehyde fixation (Amann *et al*., 1995). Fluorescence *in situ* hybridization experiments were performed as previously described (Manz *et al*., 1992). DIGA11YD11-specific oligonucleotides were tested for FISH and the best results were obtained with probe DIGA11YD11-21 (5′-TAGCATTCACCCTGAACC-3′) labelled with Cy3. This probe was used in combination with a mixture of probe Eub338-I, II and III (labelled with FITC) which detects most bacterial divisions (Daims *et al*., 1999). The Cy3-labelled nonsense probe DIGA11YD11-21 was used as a negative control. SYTO 9 (Molecular Probes) was used to stain total biomass. An inverted Zeiss confocal laser scanning microscope (CLSM, LSM510-META), equipped with three lasers (Argon 488 nm, Helium-Neon 543 nm, Helium-Neon 633 nm), was used for recording probe-conferred fluorescence signals.

### rRNA secondary structure construction

Secondary structure of 16S rRNA of the DIGA11YD11 fosmid was calculated by the crss software (P. Daegelen, unpublished) using the *E. coli* secondary structure as reference. The resulting secondary structure was then used as reference to build secondary structures of representative 16S rRNA for each OTU.

### Fosmid annotation

Gene prediction was conducted using the AMIGene software (Bocs *et al*., 2003). A total of 36 coding sequences was predicted and annotated (Barbe *et al*., 2004; Vallenet *et al*., 2006). Fosmid annotation is available at https://www.genoscope.cns.fr/agc/mage/wwwE3scope/.

### Nucleotide sequences accession numbers

Sequences determined in this study were deposited in the EMBL database under Accession No. CU367853 to CU367881 for sequences obtained from fosmid clones and CU392752 to CU392838 for sequences obtained from 16S rDNA clone libraries. DIGA11YD11 complete fosmid sequence was submitted under Accession No. CU367853.

## Supplementary material

The following supplementary material is available for this article online:

**Fig. S1.** Maximum-likelihood phylogenetic tree showing the relationship of the environmental WWE3-*radA* gene to representatives of bacterial and archaeal ones. Amino acid sequences were aligned using MUSCLE. The phylogenetic tree was conducted using PhyML software using the Jones–Taylor–Thornton (JTT) model of amino acid substitution. Heterogeneities between sites were estimated under a gamma law-based model of substitution and 100 bootstrap replicates were made. Only bootstrap values over 50% are shown for the internal branches. The scale bar indicates the number of amino acid substitutions per site.

This material is available as part of the online article from http://www.blackwell-synergy.com

## References

[b1] Amann RI, Ludwig W, Schleifer KH (1995). Phylogenetic identification and *in situ* detection of individual microbial cells without cultivation. Microbiol Rev.

[b2] Baker GC, Smith JJ, Cowan DA (2003). Review and re-analysis of domain-specific 16S primers. J Microbiol Methods.

[b3] Baker BJ, Tyson GW, Webb RI, Flanagan J, Hugenholtz P, Allen EE, Banfield JF (2006). Lineages of acidophilic *archaea* revealed by community genomic analysis. Science.

[b4] Barbe V, Vallenet D, Fonknechten N, Kreimeyer A, Oztas S, Labarre L (2004). Unique features revealed by the genome sequence of *Acinetobacter* sp. ADP1, a versatile and naturally transformation competent bacterium. Nucleic Acids Res.

[b5] Behrens S, Rühland C, Inácio J, Huber H, Fonseca A, Spencer-Martins I (2003). *In situ* accessibility of small-subunit rRNA of members of the domains *Bacteria*, *Archaea*, and *Eucarya* to Cy3-labeled oligonucleotide probes. Appl Environ Microbiol.

[b6] Bocs S, Cruveiller S, Vallenet D, Nuel G, Médigue C (2003). AMIGene: annotation of microbial genes. Nucleic Acids Res.

[b7] Brodersen DE, Clemons JWM, Carter AP, Wimberly BT, Ramakrishnan V (2002). Crystal structure of the 30s ribosomal subunit from *Thermus thermophilus*: structure of the proteins and their interactions with 16S RNA. J Mol Biol.

[b8] Chouari R, Le Paslier D, Daegelen P, Ginestet P, Weissenbach J, Sghir A (2003). Molecular evidence for novel planctomycete diversity in a municipal wastewater treatment plant. Appl Environ Microbiol.

[b9] Chouari R, Le Paslier D, Daegelen P, Ginestet P, Weissenbach J, Sghir A (2005a). Novel predominant archaeal and bacterial groups revealed by molecular analysis of an anaerobic sludge digester. Environ Microbiol.

[b10] Chouari R, Le Paslier D, Dauga C, Daegelen P, Weissenbach J, Sghir A (2005b). Novel major bacterial candidate division within a municipal anaerobic sludge digester. Appl Environ Microbiol.

[b11] Cole JR, Chai B, Farris RJ, Wang Q, Kulam SA, McGarrell DM (2005). The Ribosomal Database Project (RDP-II): sequences and tools for high-throughput rRNA analysis. Nucleic Acids Res.

[b12] Daims H, Brühl A, Amann R, Schleifer KH, Wagner M (1999). The domain-specific probe EUB338 is insufficient for the detection of all *Bacteria*: development and evaluation of a more comprehensive probe set. Syst Appl Microbiol.

[b13] DeSantis TZ, Hugenholtz P, Larsen N, Rojas M, Brodie EL, Keller K (2006). Greengenes, a chimera-checked 16S rDNA gene database and workbench compatible with ARB. Appl Environ Microbiol.

[b14] Eisen JA (1995). The RecA protein as a model molecule for molecular systematic studies of bacteria: comparison of trees of RecAs and 16S rRNAs from the same species. J Mol Evol.

[b15] Erkel C, Kube M, Reinhardt R, Liesack W (2006). Genome of rice cluster I archaea – the key methane producers in the rice rhizosphere. Science.

[b16] Fieseler L, Horn M, Wagner M, Hentschel U (2004). Discovery of the novel candidate phylum ‘*Poribacteria*’ in marine sponges. Appl Environ Microbiol.

[b17] Fuerst JA (2005). Intracellular compartmentation in planctomycetes. Annu Rev Microbiol.

[b18] Godon JJ, Zumstein E, Dabert P, Habouzit F, Moletta R (1997). Molecular microbial diversity of an anaerobic digestor as determined by small-subunit rDNA sequence analysis. Appl Environ Microbiol.

[b19] Guindon S, Gascuel O (2003). A simple, fast, and accurate algorithm to estimate large phylogenies by maximum likelihood. Syst Biol.

[b20] Gutell RR, Larsen N, Woese CR (1994). Lessons from an evolving rRNA: 16S and 23S rRNA structures from a comparative perspective. Microbiol Rev.

[b21] Hallam SJ, Mincer TJ, Schleper C, Preston CM, Roberts K, Richardson PM, DeLong EF (2006). Pathways of carbon assimilation and ammonia oxidation suggested by environmental genomic analyses of marine *Crenarchaeota*. PLoS Biol.

[b22] Harris JK, Kelley ST, Pace NR (2004). New perspective on uncultured bacterial phylogenetic division OP11. Appl Environ Microbiol.

[b23] Hicks RE, Amann RI, Stahl DA (1992). Dual staining of natural bacterioplankton with 4′,6-diamidino-2-phenylindole and fluorescent oligonucleotide probes targeting kingdom-level 16S rDNA sequences. Appl Environ Microbiol.

[b24] Hugenholtz P, Goebel BM, Pace NR (1998). Impact of culture-independent studies on the emerging phylogenetic view of bacterial diversity. J Bacteriol.

[b25] Lane DJ, Stackebrandt E, Goodfellow M (1991). 16S/23S rRNA sequencing. Nucleic Acid Techniques in Bacterial Systematics..

[b26] Leininger S, Urich T, Schloter M, Schwark L, Qi J, Nicol GW (2006). Archaea predominate among ammonia-oxidizing prokaryotes in soils. Nature.

[b27] Ludwig W, Schleifer KH (1999). Phylogeny of *bacteria* beyond the 16S rRNA standard. ASM News.

[b28] Ludwig W, Strunk O, Westram R, Richter L, Meier H, Yadhukumar (2004). ARB: a software environment for sequence data. Nucleic Acids Res.

[b29] Manz W, Amann R, Ludwig W, Wagner M, Schleifer KH (1992). Phylogenetic oligodeoxynucleotide probes for major subclasses of proteobacteria: problem and solution. Syst Appl Microbiol.

[b30] Olsen GJ, Pace NR, Nuell M, Kaine BP, Gupta R, Woese CR (1985). Sequence of the 16S rRNA gene from the thermoacidophilic archaebacterium *Sulfolobus solfataricus* and its evolutionary implications. J Mol Evol.

[b31] Pelletier E, Kreimeyer A, Bocs S, Rouy Z, Gyapay G, Chouari R (2008). Candidatus Cloacamonas acidaminovorans”: genome sequence reconstruction provides a first glimpse of a new bacterial division. J Bacteriol.

[b32] Posada D, Crandall KA (2001). Selecting models of nucleotide substitution: an application to human immunodeficiency virus 1 (HIV-1). Mol Biol Evol.

[b33] Rappé MS, Giovannoni SJ (2003). The uncultured microbial majority. Annu Rev Microbiol.

[b34] Rusch DB, Halpern AL, Sutton G, Heidelberg KB, Williamson S, Yooseph S (2007). The sorcerer II global ocean sampling expedition: Northwest Atlantic through Eastern Tropical Pacific. PLoS Biol.

[b35] Sandler SJ, Hugenholtz P, Schleper C, DeLong EF, Pace NR, Clark AJ (1999). Diversity of *radA* genes from cultured and uncultured archaea: comparative analysis of putative RadA proteins and their use as a phylogenetic marker. J Bacteriol.

[b36] Schuwirth BS, Borovinskaya MA, Hau CW, Zhang W, Vila-Sanjurjo A, Holton JM, Cate JH (2005). Structures of the bacterial ribosome at 3.5 A resolution. Science.

[b37] Stackebrandt E, Goebel BM (1994). Taxonomic note: a place for DNA–DNA reassociation and 16S rRNA sequence-analysis in the present species definition in bacteriology. Int J Syst Bacteriol.

[b38] Swofford DL (2002). PAUP*: Phylogenetic Analysis Using Parsimony (and Other Methods) Version 4.0.

[b39] Tavaré S, Miura RM (1986). Some probabilistic and statistical problems in the analysis of DNA sequences. Some Mathematical Questions in Biology – DNA Sequence Analysis.

[b40] Treusch AH, Leininger S, Kletzin A, Schuster SC, Klenk H-P, Schleper C (2005). Novel genes for nitrite reductase and Amo-related proteins indicate a role of uncultivated mesophilic crenarchaeota in nitrogen cycling. Environ Microbiol.

[b41] Tyson GW, Chapman J, Hugenholtz P, Allen EE, Ram RJ, Richardson PM (2004). Community structure and metabolism through reconstruction of microbial genomes from the environment. Nature.

[b42] Vallenet D, Labarre L, Rouy Z, Barbe V, Bocs S, Cruveiller S (2006). MaGe: a microbial genome annotation system supported by synteny results. Nucleic Acids Res.

[b43] Venter JC, Remington K, Heidelberg JF, Halpern AL, Rusch D, Eisen JA (2004). Environmental genome shotgun sequencing of the Sargasso Sea. Science.

[b44] Weisburg WG, Barns SM, Pelletier DA, Lane DJ (1991). 16S ribosomal DNA amplification for phylogenetic study. J Bacteriol.

[b45] Woese CR (1987). Bacterial evolution. Microbiol Rev.

[b46] Wu XL, Friedrich MW, Conrad R (2006). Diversity and ubiquity of thermophilic methanogenic archaea in temperate anoxic soils. Environ Microbiol.

[b47] Zheng D, Alm EW, Stahl DA, Raskin L (1996). Characterization of universal small-subunit rRNA hybridization probes for quantitative molecular microbial ecology studies. Appl Environ Microbiol.

