# Supplementary figures and images for: Discovery and characterization of a new bacterial candidate division by an anaerobic sludge digester metagenomic approach
Source: Environ Microbiol. 2008 Aug;10(8):2111–23. doi: 10.1111/j.1462-2920.2008.01632.x (PMC2702496; doi:10.1111/j.1462-2920.2008.01632.x)

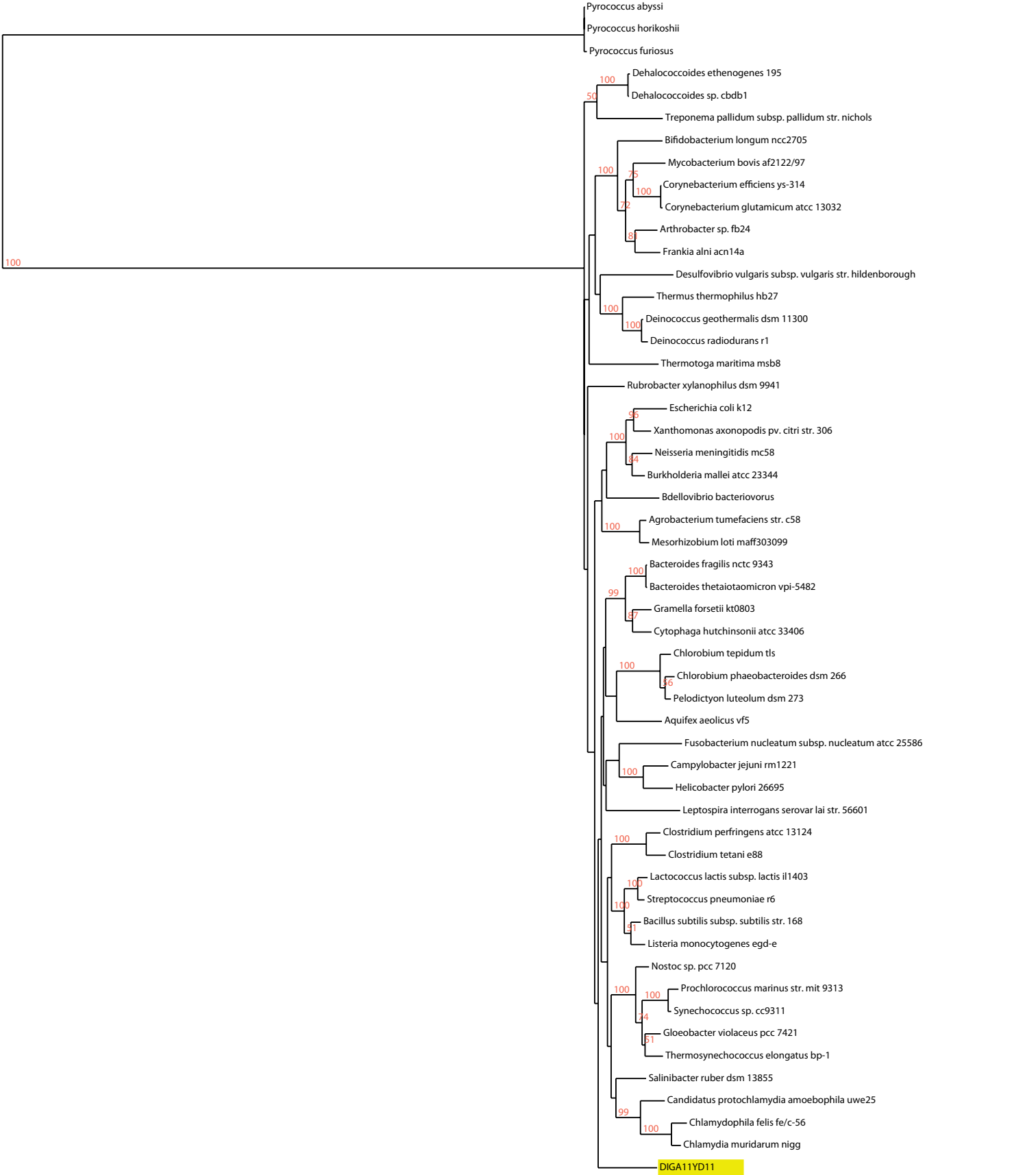

Supplement: Supplementary file 1 [file emi0010-2111-SD1.pdf]
